# Aromaticine, a sesquiterpene lactone from *Amblyopappus pusillus*

**DOI:** 10.1107/S1600536808002729

**Published:** 2008-01-30

**Authors:** Iván Brito, Jorge Bórquez, Luis Alberto Loyola, Matías López-Rodríguez

**Affiliations:** a Departamento de Química, Facultad de Ciencias Básicas, Universidad de Antofagasta, Casilla 170, Antofagasta, Chile; b Instituto de Bio-Orgánica ’Antonio González’, Universidad de La Laguna, Astrofísico Francisco Sánchez No. 2, La Laguna, Tenerife, Spain

## Abstract

Aromaticine (systematic name: 4a,8-dimethyl-3-methyl­ene-3,3a,4,4a,7a,8,9,9a-octa­hydro­azuleno[6,5-*b*]furan-2,5-dione), C_15_H_18_O_3_, is a natural lactone isolated from *Amblyopappus pusillus*. The mol­ecular structure and conformation agree with the results of Romo, Joseph-Nathan & Díaz [(1964 ▶). *Tetra­hedron*, **20**, 79–85]. The fused-ring system contains a seven-membered ring in a twist-boat conformation and two five-membered rings *trans* fused in envelope conformations.

## Related literature

For related literature, see: Allen *et al.* (1987 ▶); Bórquez (2006 ▶); Bernstein *et al.* (1995 ▶); Cremer & Pople (1975 ▶); Rodríguez *et al.* (1976 ▶); Romo *et al.* (1964 ▶).
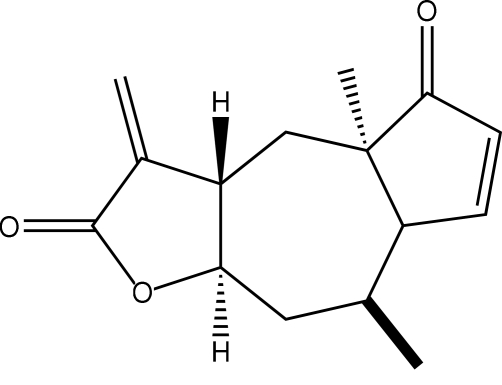


## Experimental

### 

#### Crystal data

C_15_H_18_O_3_
*M* *_r_* = 246.29
Orthorhombic, 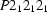

*a* = 6.763 (4) Å
*b* = 9.932 (5) Å
*c* = 18.685 (7) Å
*V* = 1255.1 (11) Å^3^
*Z* = 4
Mo *K*α radiation
μ = 0.09 mm^−1^
*T* = 295 (2) K
0.40 × 0.10 × 0.08 mm

#### Data collection

Nonius KappaCCD area-detector diffractometer
Absorption correction: none
16315 measured reflections
1667 independent reflections
1432 reflections with *I* > 2σ(*I*)
*R*_int_ = 0.051

#### Refinement

*R*[*F* ^2^ > 2σ(*F* ^2^)] = 0.057
*wR*(*F* ^2^) = 0.148
*S* = 1.20
1667 reflections
167 parameters
H-atom parameters constrained
Δρ_max_ = 0.24 e Å^−3^
Δρ_min_ = −0.23 e Å^−3^

### 

Data collection: *COLLECT* (Nonius, 1998 ▶); cell refinement: *DENZO-SMN* (Otwinowski & Minor, 1997 ▶); data reduction: *DENZO-SMN*; program(s) used to solve structure: *SIR97* (Altomare *et al.*, 1999 ▶); program(s) used to refine structure: *SHELXL97* (Sheldrick, 2008 ▶); molecular graphics: *ORTEP-3 for Windows* (Farrugia, 1997 ▶) and *PLATON* (Spek, 2003 ▶); software used to prepare material for publication: *WinGX* (Farrugia, 1999 ▶).

## Supplementary Material

Crystal structure: contains datablocks global, I. DOI: 10.1107/S1600536808002729/om2203sup1.cif

Structure factors: contains datablocks I. DOI: 10.1107/S1600536808002729/om2203Isup2.hkl

Additional supplementary materials:  crystallographic information; 3D view; checkCIF report

## Figures and Tables

**Fig. 1.**
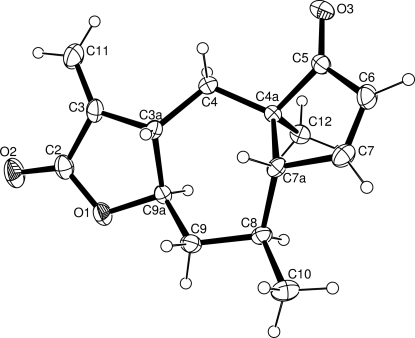
View of the title compound showing the atom-numbering scheme. Displacement ellipsoids are drawn at the 30% probability level, and H-atom radii are arbitrary.

**Fig. 2.**
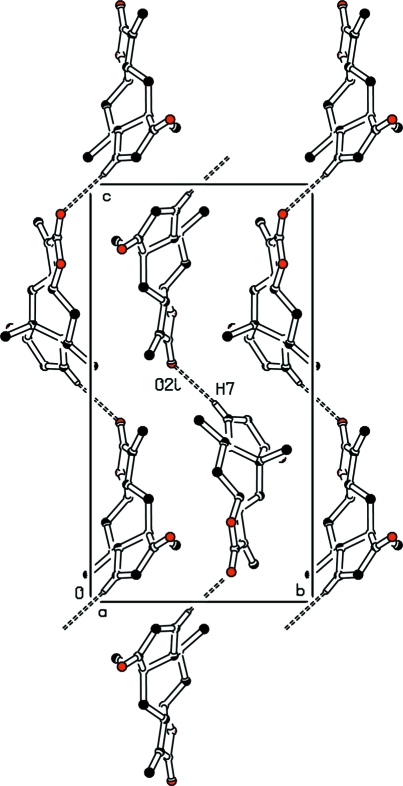
Part of the crystal structure showing the formation of C(9) along [001]. For the sake of clarity, H atoms not involved in hydrogen bonding have been omitted. [Symmetry code: (i) 1/2 - x, 1 - y, 1/2 + z.]

## supplementary crystallographic information

### Comment

Sesquiterpene lactones are known to have biological activity, and some guaianolides have antitumor and citotoxic activities (Rodríguez *et al.*, 1976). The title compound was isolated from aerial parts of Amblyopappus pusillus (Bórquez, 2006), which was originally isolated from Helenium Aromaticum (Romo *et al.*, 1964).In order to ascertain the structure and secure the assignment of the stereochemistry, an X-ray structure determination was performed. The absolute configuration was not determined. The structure confirms the previously proposed molecular structure and molecular conformation (Romo *et al.*, 1964). The molecule (Fig. 1) involves a seven-membered ring in a twisted- boat conformation. Deviations of atoms in this ring from the plane (Cremer & Pople, 1975) were less than 0.1 Å for atoms C4 and C9 and more than 0.38 Å, for the remaining atoms of the ring. Using the same definition, for the two five-membered rings, the lactone is present in an envelope conformation with C3a in the flap position and a maximun deviation of -0.144 (3) Å for C3a. The other five-membered ring also adopts an envelope conformation, with C4a 0.137 (3) Å out of the plane. The crystal structure consists of discrete molecules. The molecules are linked into chains by one weak intermolecular C—H···O hydrogen bond [H···O =2.65 Å, C···O = 3.552 (4) Å and C—H···O =163°]. Atom C7 acts as a hydrogen bond donor *via* atom H7 to atom O2 in the molecule at (-*x* + 1/2,-*y* + 1, *z* + 1/2), so generating a C(9) chain running parallel to the [001] direction, (Bernstein, *et al.*, 1995). Bond lengths are within expected ranges (Allen *et al.*, 1987), with average values (Å): O-C*sp*^2 ^=1.205 (4); C*sp*^2^—C*sp*^2 ^= 1.440 (5); C*sp*^3^—C*sp*^2 ^=1.501 (5) and C*sp*^3^—C*sp*^3^= 1.516 (4).

### Experimental

The whole fresh plants (200 g) was submerged in chloroform at room temperature for 4 hrs. After filtration, the solvent was avaporated to dryness under reduced pressure, yielding 10 g. The chloroform extract was chromatographed on silica gel column using diethyl ether, giving 800 mg of the title compound (m.p 505–527 K). Optical rotation: [α] _D_^20 ^+20.6. The title compound was identified by comparing the spectroscopic data with the previously published data (Romo *et al.*, 1964). Crystal suitable for X-ray analysis were obtained for recrystallization from diethyl ether at room temperature.

### Refinement

H atoms were located from difference Fourier maps and placed in geometrically idealized positions (C—H = 0.93–0.98 Å), and were constrained to ride on their parents atoms with *U*_iso_ (H) = 1.5*U*_eq_(C) for methyl H atoms and 1.2 *U*_eq_(C) for the other H atoms.

### Crystal data

**Table:**

C_15_H_18_O_3_	*F*_000_ = 528
*M**_r_* = 246.29	*D*_x_ = 1.303 Mg m^−^^3^
Orthorhombic, *P*2_1_2_1_2_1_	Mo *K*α radiation λ = 0.7107 Å
Hall symbol: P 2ac 2ab	Cell parameters from 1595 reflections
*a* = 6.763 (4) Å	θ = 2.3–27.5º
*b* = 9.932 (5) Å	µ = 0.09 mm^−^^1^
*c* = 18.685 (7) Å	*T* = 295 (2) K
*V* = 1255.1 (11) Å^3^	Needle, colourless
*Z* = 4	0.40 × 0.10 × 0.08 mm

### Data collection

**Table:**

Nonius KappaCCD area-detector diffractometer	1432 reflections with *I* > 2σ(*I*)
Radiation source: fine-focus sealed tube	*R*_int_ = 0.051
Monochromator: graphite	θ_max_ = 27.5º
φ and ω scans with κ offsets	θ_min_ = 2.3º
Absorption correction: none	*h* = −8→8
16315 measured reflections	*k* = −12→12
1667 independent reflections	*l* = −22→24

### Refinement

**Table:**

Refinement on *F*^2^	H-atom parameters constrained
Least-squares matrix: full	*w* = 1/[σ^2^(*F*_o_^2^) + (0.0749*P*)^2^ + 0.1397*P*] where *P* = (*F*_o_^2^ + 2*F*_c_^2^)/3
*R*[*F*^2^ > 2σ(*F*^2^)] = 0.057	(Δ/σ)_max_ < 0.001
*wR*(*F*^2^) = 0.148	Δρ_max_ = 0.24 e Å^−^^3^
*S* = 1.20	Δρ_min_ = −0.23 e Å^−^^3^
1667 reflections	Extinction correction: none
167 parameters	

### Special details

**Table:**

Geometry. All e.s.d.'s (except the e.s.d. in the dihedral angle between two l.s. planes) are estimated using the full covariance matrix. The cell e.s.d.'s are taken into account individually in the estimation of e.s.d.'s in distances, angles and torsion angles; correlations between e.s.d.'s in cell parameters are only used when they are defined by crystal symmetry. An approximate (isotropic) treatment of cell e.s.d.'s is used for estimating e.s.d.'s involving l.s. planes.
Refinement. Refinement of *F*^2^ against ALL reflections. The weighted *R*-factor *wR* and goodness of fit *S* are based on *F*^2^, conventional *R*-factors *R* are based on *F*, with *F* set to zero for negative *F*^2^. The threshold expression of *F*^2^ > σ(*F*^2^) is used only for calculating *R*-factors(gt) *etc*. and is not relevant to the choice of reflections for refinement. *R*-factors based on *F*^2^ are statistically about twice as large as those based on *F*, and *R*- factors based on ALL data will be even larger.

### Fractional atomic coordinates and isotropic or equivalent isotropic displacement parameters (Å^2^)

**Table:**

	*x*	*y*	*z*	*U*_iso_*/*U*_eq_	
O1	0.5372 (3)	0.6338 (2)	0.19079 (12)	0.0407 (6)	
O2	0.5286 (4)	0.6343 (3)	0.07133 (13)	0.0575 (8)	
O3	−0.2760 (3)	0.8586 (3)	0.34464 (13)	0.0411 (6)	
C2	0.4451 (5)	0.6506 (3)	0.12748 (18)	0.0388 (8)	
C3	0.2351 (5)	0.6883 (3)	0.14092 (17)	0.0352 (8)	
C3A	0.1973 (4)	0.6661 (3)	0.21918 (14)	0.0266 (7)	
H3A	0.147	0.5742	0.2248	0.032 (3)*	
C4	0.0484 (4)	0.7610 (3)	0.25401 (15)	0.0327 (7)	
H4A	−0.0835	0.7355	0.2388	0.032 (3)*	
H4B	0.0728	0.8517	0.2369	0.032 (3)*	
C4A	0.0554 (4)	0.7616 (3)	0.33662 (15)	0.0246 (6)	
C5	−0.1505 (4)	0.7855 (3)	0.36921 (16)	0.0290 (7)	
C6	−0.1602 (5)	0.7054 (4)	0.43487 (17)	0.0398 (8)	
H6	−0.257	0.7141	0.4699	0.032 (3)*	
C7	−0.0114 (4)	0.6190 (3)	0.43729 (17)	0.0362 (8)	
H7	0.0105	0.5602	0.4752	0.032 (3)*	
C7A	0.1190 (4)	0.6263 (3)	0.37165 (15)	0.0270 (7)	
H7A	0.0729	0.555	0.3394	0.032 (3)*	
C8	0.3431 (4)	0.6025 (3)	0.38199 (16)	0.0294 (7)	
H8	0.401	0.6854	0.4012	0.032 (3)*	
C9	0.4455 (5)	0.5710 (3)	0.31060 (17)	0.0335 (7)	
H9A	0.5868	0.5666	0.319	0.032 (3)*	
H9B	0.4032	0.4824	0.295	0.032 (3)*	
C9A	0.4083 (4)	0.6694 (3)	0.25025 (15)	0.0301 (7)	
H9C	0.4391	0.7608	0.2665	0.032 (3)*	
C10	0.3856 (5)	0.4889 (4)	0.43484 (19)	0.0461 (9)	
H10A	0.3261	0.4071	0.4179	0.045 (4)*	
H10B	0.3315	0.5116	0.4808	0.045 (4)*	
H10C	0.5259	0.4766	0.4391	0.045 (4)*	
C11	0.1178 (6)	0.7350 (4)	0.09104 (18)	0.0545 (10)	
H11A	0.1645	0.7459	0.0446	0.065*	
H11B	−0.0123	0.7572	0.102	0.065*	
C12	0.1774 (5)	0.8833 (3)	0.36430 (17)	0.0316 (7)	
H12A	0.1202	0.9654	0.3468	0.045 (4)*	
H12B	0.3112	0.876	0.3475	0.045 (4)*	
H12C	0.1764	0.8838	0.4157	0.045 (4)*	

### Atomic displacement parameters (Å^2^)

**Table:**

	*U*^11^	*U*^22^	*U*^33^	*U*^12^	*U*^13^	*U*^23^
O1	0.0379 (12)	0.0473 (14)	0.0370 (13)	0.0043 (12)	0.0092 (10)	−0.0058 (11)
O2	0.0744 (17)	0.0583 (17)	0.0399 (14)	0.0075 (16)	0.0252 (13)	0.0018 (13)
O3	0.0294 (11)	0.0432 (14)	0.0507 (14)	0.0076 (11)	0.0000 (11)	−0.0008 (12)
C2	0.0501 (19)	0.0310 (18)	0.0353 (19)	0.0002 (16)	0.0102 (15)	0.0002 (15)
C3	0.0500 (19)	0.0281 (17)	0.0274 (17)	0.0009 (15)	0.0029 (14)	−0.0045 (14)
C3A	0.0305 (14)	0.0260 (16)	0.0233 (15)	−0.0010 (13)	−0.0010 (11)	−0.0018 (13)
C4	0.0326 (16)	0.0393 (18)	0.0261 (15)	0.0081 (14)	−0.0029 (12)	0.0022 (14)
C4A	0.0240 (13)	0.0245 (15)	0.0252 (14)	0.0024 (12)	0.0008 (11)	−0.0011 (12)
C5	0.0255 (14)	0.0283 (17)	0.0330 (17)	−0.0014 (13)	−0.0005 (12)	−0.0081 (13)
C6	0.0352 (16)	0.054 (2)	0.0303 (17)	−0.0053 (16)	0.0080 (13)	0.0012 (16)
C7	0.0379 (16)	0.0404 (19)	0.0304 (17)	−0.0079 (15)	−0.0037 (13)	0.0074 (15)
C7A	0.0319 (15)	0.0249 (16)	0.0242 (15)	−0.0043 (12)	−0.0046 (12)	0.0003 (13)
C8	0.0327 (15)	0.0254 (16)	0.0300 (16)	0.0023 (13)	−0.0054 (12)	0.0027 (13)
C9	0.0312 (15)	0.0315 (17)	0.0378 (18)	0.0063 (14)	−0.0044 (14)	−0.0028 (14)
C9A	0.0296 (14)	0.0312 (17)	0.0295 (16)	0.0037 (12)	0.0026 (12)	−0.0045 (14)
C10	0.047 (2)	0.043 (2)	0.048 (2)	0.0062 (17)	−0.0088 (18)	0.0134 (18)
C11	0.073 (3)	0.063 (3)	0.0277 (18)	0.017 (2)	−0.0028 (17)	0.0011 (18)
C12	0.0318 (15)	0.0259 (17)	0.0369 (18)	−0.0006 (13)	0.0002 (13)	0.0009 (14)

### Geometric parameters (Å, °)

**Table:**

O1—C2	1.347 (4)	C7—C7A	1.512 (4)
O1—C9A	1.456 (3)	C7—H7	0.93
O2—C2	1.202 (4)	C7A—C8	1.546 (4)
O3—C5	1.208 (4)	C7A—H7A	0.98
C2—C3	1.490 (5)	C8—C10	1.526 (4)
C3—C11	1.309 (5)	C8—C9	1.535 (4)
C3—C3A	1.501 (4)	C8—H8	0.98
C3A—C4	1.525 (4)	C9—C9A	1.513 (4)
C3A—C9A	1.541 (4)	C9—H9A	0.97
C3A—H3A	0.98	C9—H9B	0.97
C4—C4A	1.544 (4)	C9A—H9C	0.98
C4—H4A	0.97	C10—H10A	0.96
C4—H4B	0.97	C10—H10B	0.96
C4A—C5	1.538 (4)	C10—H10C	0.96
C4A—C12	1.552 (4)	C11—H11A	0.93
C4A—C7A	1.555 (4)	C11—H11B	0.93
C5—C6	1.463 (5)	C12—H12A	0.96
C6—C7	1.323 (5)	C12—H12B	0.96
C6—H6	0.93	C12—H12C	0.96
			
C2—O1—C9A	111.3 (2)	C7—C7A—H7A	106.1
O2—C2—O1	122.2 (3)	C8—C7A—H7A	106.1
O2—C2—C3	128.9 (3)	C4A—C7A—H7A	106.1
O1—C2—C3	108.9 (3)	C10—C8—C9	109.1 (3)
C11—C3—C2	123.2 (3)	C10—C8—C7A	112.2 (3)
C11—C3—C3A	130.0 (3)	C9—C8—C7A	111.4 (2)
C2—C3—C3A	106.8 (3)	C10—C8—H8	108
C3—C3A—C4	115.9 (3)	C9—C8—H8	108
C3—C3A—C9A	101.9 (2)	C7A—C8—H8	108
C4—C3A—C9A	116.0 (2)	C9A—C9—C8	116.2 (2)
C3—C3A—H3A	107.5	C9A—C9—H9A	108.2
C4—C3A—H3A	107.5	C8—C9—H9A	108.2
C9A—C3A—H3A	107.5	C9A—C9—H9B	108.2
C3A—C4—C4A	114.1 (2)	C8—C9—H9B	108.2
C3A—C4—H4A	108.7	H9A—C9—H9B	107.4
C4A—C4—H4A	108.7	O1—C9A—C9	108.2 (2)
C3A—C4—H4B	108.7	O1—C9A—C3A	105.2 (2)
C4A—C4—H4B	108.7	C9—C9A—C3A	114.9 (3)
H4A—C4—H4B	107.6	O1—C9A—H9C	109.4
C5—C4A—C4	111.6 (2)	C9—C9A—H9C	109.4
C5—C4A—C12	103.2 (2)	C3A—C9A—H9C	109.4
C4—C4A—C12	110.6 (2)	C8—C10—H10A	109.5
C5—C4A—C7A	102.5 (2)	C8—C10—H10B	109.5
C4—C4A—C7A	115.2 (2)	H10A—C10—H10B	109.5
C12—C4A—C7A	112.7 (2)	C8—C10—H10C	109.5
O3—C5—C6	127.9 (3)	H10A—C10—H10C	109.5
O3—C5—C4A	125.4 (3)	H10B—C10—H10C	109.5
C6—C5—C4A	106.8 (3)	C3—C11—H11A	120
C7—C6—C5	110.3 (3)	C3—C11—H11B	120
C7—C6—H6	124.8	H11A—C11—H11B	120
C5—C6—H6	124.8	C4A—C12—H12A	109.5
C6—C7—C7A	112.6 (3)	C4A—C12—H12B	109.5
C6—C7—H7	123.7	H12A—C12—H12B	109.5
C7A—C7—H7	123.7	C4A—C12—H12C	109.5
C7—C7A—C8	117.6 (2)	H12A—C12—H12C	109.5
C7—C7A—C4A	102.8 (2)	H12B—C12—H12C	109.5
C8—C7A—C4A	117.1 (2)		
			
C9A—O1—C2—O2	175.7 (3)	C5—C6—C7—C7A	−1.4 (4)
C9A—O1—C2—C3	−5.1 (4)	C6—C7—C7A—C8	145.1 (3)
O2—C2—C3—C11	−13.6 (6)	C6—C7—C7A—C4A	14.8 (3)
O1—C2—C3—C11	167.1 (3)	C5—C4A—C7A—C7	−20.9 (3)
O2—C2—C3—C3A	168.1 (4)	C4—C4A—C7A—C7	−142.3 (3)
O1—C2—C3—C3A	−11.1 (4)	C12—C4A—C7A—C7	89.5 (3)
C11—C3—C3A—C4	−30.1 (5)	C5—C4A—C7A—C8	−151.4 (3)
C2—C3—C3A—C4	148.0 (3)	C4—C4A—C7A—C8	87.2 (3)
C11—C3—C3A—C9A	−157.0 (4)	C12—C4A—C7A—C8	−41.0 (4)
C2—C3—C3A—C9A	21.1 (3)	C7—C7A—C8—C10	40.3 (4)
C3—C3A—C4—C4A	−164.1 (3)	C4A—C7A—C8—C10	163.6 (3)
C9A—C3A—C4—C4A	−44.6 (4)	C7—C7A—C8—C9	163.0 (3)
C3A—C4—C4A—C5	−147.0 (3)	C4A—C7A—C8—C9	−73.8 (3)
C3A—C4—C4A—C12	98.7 (3)	C10—C8—C9—C9A	176.3 (3)
C3A—C4—C4A—C7A	−30.6 (4)	C7A—C8—C9—C9A	51.9 (4)
C4—C4A—C5—O3	−36.1 (4)	C2—O1—C9A—C9	142.0 (3)
C12—C4A—C5—O3	82.7 (3)	C2—O1—C9A—C3A	18.7 (3)
C7A—C4A—C5—O3	−160.0 (3)	C8—C9—C9A—O1	172.3 (2)
C4—C4A—C5—C6	144.9 (3)	C8—C9—C9A—C3A	−70.5 (4)
C12—C4A—C5—C6	−96.3 (3)	C3—C3A—C9A—O1	−23.8 (3)
C7A—C4A—C5—C6	21.0 (3)	C4—C3A—C9A—O1	−150.6 (3)
O3—C5—C6—C7	168.1 (3)	C3—C3A—C9A—C9	−142.7 (3)
C4A—C5—C6—C7	−13.0 (4)	C4—C3A—C9A—C9	90.5 (3)
